# Dabigatran: patient management in specific clinical settings

**DOI:** 10.1007/s00508-014-0581-x

**Published:** 2014-08-20

**Authors:** Paul Alexander Kyrle, Konrad Binder, Sabine Eichinger, Reinhold Függer, Bernd Gollackner, J. Michael Hiesmayr, Kurt Huber, Wielfried Lang, Peter Perger, Peter Quehenberger, Franz X. Roithinger, Sabine Schmaldienst, Ansgar Weltermann, Hans Domanovits

**Affiliations:** 1 Department of Medicine I, Medical University of Vienna, Waehringer Guertel 18–20, 1090 Vienna, Austria; 2 Department of Cardiosurgery, Landesklinikum St. Pölten, Sankt Pölten, Austria; 3 Department of Surgery, Elisabethinen Hospital Linz, Linz, Austria; 4 Department of Surgery, Division of Vascular Surgery, Medical University of Vienna, Vienna, Austria; 5 Department of Cardiothoracic and Vascular Anaesthesia and Critical Care Medicine, Medical University of Vienna, Vienna, Austria; 6 3rd Medical Department, Cardiology and Intensive Care Unit III, 3rd Medical Department, Wilhelminenspital, Vienna, Austria; 7 Department of Neurology, Spital der Barmherzigen Brüder, Vienna, Austria; 8 Department of Anaesthesia and Critical Care, Krankenhaus Hietzing and Neurologisches Zentrum Rosenhügel, Vienna, Austria; 9 Department of Laboratory Medicine, Medical University of Vienna, Vienna, Austria; 10 Department of Internal Medicine, Landesklinikum Baden Mödling, Mödling, Austria; 11 Department of Medicine III, Division of Clinical Nephrology and Dialysis, Medical University of Vienna, Vienna, Austria; 12 Department of Medicine I, Elisabethinen Hospital Linz, Linz, Austria; 13 University Clinic for Emergency Medicine, Medical University of Vienna, Vienna, Austria

**Keywords:** Dabigatran, Bleeding, Surgery, Acute coronary syndrome, Stroke, Dabigatran, Blutung, Operation, Akutes Koronarsyndrom, Schlaganfall

## Abstract

Dabigatran, a direct thrombin inhibitor, is licensed for the prevention of venous thromboembolism after knee and hip replacement, the prevention of stroke and systemic embolism in patients with non-valvular atrial fibrillation and for the treatment of acute venous thromboembolism. As dabigatran has a favourable benefit–risk profile, it is being increasingly used. Dabigatran differs from vitamin K antagonists as regards its pharmacological characteristics and its impact on certain laboratory tests, and also in the lack of a direct antagonist that can reverse dabigatran-induced anticoagulation. In emergency settings such as acute bleeding, emergency surgery, acute coronary syndrome, thrombolysis for ischaemic stroke or overdosing, specific strategies are required. A working group of experts from various disciplines has developed strategies for the management of dabigatran-treated patients in emergency settings.

## Abstract (de)

Dabigatran, ein direkter Thrombin-Inhibitor, ist zur Prophylaxe venöser Thromboembolien nach Knie- oder Hüftgelenkersatz, zur Prophylaxe von Schlaganfällen und systemischer Embolien bei nicht-valvulärem Vorhofflimmern zugelassen und zur Therapie der akuten venösen Thromboembolie. Aufgrund des günstigen Nutzen/Risiko-Profils wird eine zunehmende Zahl von Patienten mit Dabigatran behandelt. Dabigatran unterscheidet sich von den Vitamin K-Antagonisten durch seine pharmakologischen Eigenschaften, seine Beeinflussung von Labortests, aber auch durch fehlende Antagonisierbarkeit. In Notfallsituationen, wie beispielsweise bei einer akuten Blutung, einer Notoperation, einem akuten Koronarsyndrom, der Thrombolyse bei ischämischem Schlaganfall oder einer Überdosierung, sind spezielle Strategien erforderlich. Eine Arbeitsgruppe, die sich aus Experten unterschiedlicher Fachrichtungen zusammensetzt, hat Therapiestrategien zum Management akuter Notfallsituationen bei Patienten, die mit Dabigatran behandelt werden, erarbeitet.

## Background

Dabigatran has been approved for primary prevention of venous thromboembolism in adult patients after elective hip or knee replacement, for the prevention of stroke and systemic embolism in patients with non-valvular atrial fibrillation, and for the treatment of acute venous thromboembolism. Due to its simpler management and its favourable risk/benefit profile compared with vitamin K antagonists, the number of dabigatran-treated patients is steadily increasing.

As with any drug that inhibits coagulation, bleeding can also occur with dabigatran. At present, no direct antidotes that can specifically reverse the effects of dabigatran are available, even though there is intense research going on. In this report, strategies for the management of acute bleeding, either spontaneously or in the context of surgery or injury, are addressed. In addition, management strategies for specific acute clinical settings including ischaemic stroke, acute coronary syndrome and overdosing are discussed. The recommendations relate exclusively to the specific dabigatran effects and are based on a very low level of scientific evidence. General measures that are also applied in non-dabigatran-treated patients in the above-mentioned settings will not be discussed. The general management of bleeding should be performed according to guidelines of national and international societies or local institutions. In this context, we refer to recent publications [1–4].

## Overview of phase III studies

### Knee or hip replacement

Dabigatran 150 mg or 220 mg once daily (OD) is as effective as enoxaparin 40 mg OD or 30 mg twice (BID) daily in the prevention of postsurgical venous thromboembolism. Dabigatran and enoxaparin treatments also confer comparable risks of severe bleeding (RR 0.72 (0.27–1.89), *p* = 0.50; 0.73(0.38–1.40), *p* = 0.34, respectively) [5].

### Acute venous thromboembolism

In the treatment of acute venous thromboembolism enoxaparin followed by dabigatran 150 mg BID is as effective and safe as enoxaparin followed by a vitamin K antagonist (incidence of recurrent venous thromboembolism: 2.4 % vs 2.1 %; HR 1.1 (0.65–1.82); incidence of major bleeding: 1.6 % vs 1.9 %; HR 0.82 (0.45–1.48)) [6].

### Non-valvular atrial fibrillation

For the prevention of stroke or systemic embolism, treatment with dabigatran 150 mg BID is significantly more effective than warfarin (annual incidences: 1.11 % vs 1.71 %; *p* < 0.001). The lower dose of dabigatran (110 mg BID) is equally effective as warfarin (annual incidences: 1.54 % vs 1.71 %). The risk of severe bleeding is similar in patients treated with the high dose of dabigatran dose as compared with warfarin therapy (3.32 % vs 3.57 %) and is significantly lower with the lower dose of dabigatran (2.87 % vs 3.57 %). Intracranial bleeding was observed significantly less often with dabigatran than with warfarin (high dose: 0.31 %; low dose: 0.23 %; warfarin: 0.76 %; *p* < 0.001). Fatal intracranial bleeding occurred infrequently (dabigatran 150 mg BID: 13 cases, dabigatran 110 mg BID: 11 cases; warfarin: 32 cases) Mortality due to intracranial bleeding was similar across all treatment groups (35 %, 41 % and 36 %, respectively). Gastrointestinal bleeding occurred more frequently with dabigatran 150 mg BID than with warfarin (annual incidences: 1.56 % vs 1.07 %) [7–10].

In five phase III studies approximately 27,500 patients were treated with either dabigatran or warfarin over 6–36 months [11]. The 30-day mortality due to severe bleeding was 9 % with dabigatran and 13 % with warfarin (odds ratio 0.66 (0.44–1.0); *p* = 0.051). Overall, 1162 cases of severe bleeding occurred with dabigatran or warfarin treatment. Only 10 of these patients were treated with prothrombin complex concentrates (PCC) and 11 patients received recombinant factor VIIa (rFVIIa). Only 1 patient underwent haemodialysis. These small patient numbers preclude conclusions on the efficacy of these therapies.

## Pharmacological features

Dabigatran etexilate is administered orally and is rapidly hydrolysed to the active drug dabigatran. Dabigatran inhibits directly and reversibly both free and clot-bound thrombin. The peak dabigatran plasma level is reached approximately 2 h after ingestion. Allowing for the considerable inter-individual variety, these peak plasma levels reach, on average, 175 ng/mL in the context of long-term dabigatran administration at a dose of 150 mg BID. The trough levels are 91 ng/mL after 12 h [12]. Approximately 80 % of dabigatran is excreted unaltered via the kidneys. The half-life of dabigatran in plasma depends on the kidney function: at a creatinine clearance (CrCl) of greater than 80 mL/min, 50–80 mL/min or 30–50 mL/min, the half-life is 13 h, 15 h and 18 h, respectively, and increases to 27 h at a CrCl of less than 30 mL/min. Due to low protein binding, dabigatran is dialyzable. P-glycoprotein inhibitors such as amiodarone, verapamil, cyclosporine or ketoconazole increase dabigatran plasma levels, while P-glycoprotein inducers such as rifampicine and carbamazepin have the opposite effect.

### Effects on laboratory tests

Dabigatran affects all coagulation and thrombin-dependent platelet function tests [13]. The extent depends on the dose, the time interval between dabigatran ingestion and blood sampling and the kidney function. Treatment with dabigatran does not routinely require laboratory monitoring. In the case of bleeding, in the peri-operative setting, or prior to major surgery or invasive procedure, coagulation tests could be helpful. The choice of test for the estimation of a dabigatran effect depends on availability, linearity of the dose-effect curve and standardisation. Assessment of thrombin activity using a chromogenic substrate or the ecarin clotting time is not addressed, as these test systems are only available in specialised coagulation laboratories.

The thrombin clotting time (TT) measures the conversion of fibrinogen to fibrin, which is the final step in the coagulation cascade. The TT is prolonged in the presence of thrombin inhibitors such as dabigatran. The TT prolongation is directly proportional to the dabigatran concentration. At a dabigatran plasma concentration of 200 ng/mL (peak level), the TT is approximately 15-fold prolonged. A normal TT value precludes a clinically relevant dabigatran level. Conversely, a prolonged TT can be indicative for (residual) dabigatran activity. However, the extent of a potential bleeding risk cannot be deduced from TT prolongation. The plasma concentration of dabigatran can also be measured by calibrated TTs, such as the Hemoclot test system*.* The lower detection limit is 20 ng/mL [14, 15].

Dabigatran also prolongs the activated partial thromboplastin time (aPTT). At a dabigatran level of 200 ng/mL, the aPTT is of approximately 2.5-fold prolonged. The dose-effect curve flattens at higher dabigatran levels. The magnitude of the effect of dabigatran on the aPTT depends on the reagent. A normal aPTT does not fully preclude a clinically relevant dabigatran level [16], but is commonly accepted as evidence for a lack of anticoagulatory effects of dabigatran in the peri-interventional setting.

Both the prothrombin time and the activated clotting time can be prolonged during dabigatran treatment. The sensitivity of the prothrombin time towards dabigatran is low and substantial differences between various prothrombin time reagents have been found [17]. The low sensitivity of the activated clotting time restricts its use under routine conditions [18].

### Reversal

Data from animal and in-vitro experiments will not be discussed, because they are irrelevant from a clinical point of view. An antidote that can antagonise the effects of dabigatran, idarucizumab, has been shown to be safe in a phase I study [19].

Dabigatran is lipophilic and binds to activated charcoal. A 57-year-old suicidal woman ingested 11 g dabigatran and survived after she was treated with activated charcoal (plus gastric lavage) [20]. Due to the rapid absorption of dabigatran, the administration of activated charcoal is only reasonable within the first 2 (to 4) h after ingestion.

Dabigatran is dialysable and several case reports show that haemodialysis is effective prior to emergency surgery [20] or in the case of life-threatening bleeding [22, 23]. Approximately two thirds of dabigatran can be removed from the circulation within 4 h. The practical aspects as regards haemodialysis are summarised in Table 1.

**Table 1:** Haemodialysis in dabigatran-treated patients

Procedure, duration	Intermittent: haemodialysis Continuous haemofiltration (in unstable patients): 3–4 h each
Access	Haemofiltration catheter
Dialysis membrane	In haemodialysis: mid-flux or high-flux membranes (there are currently no data for low-flux membranes)
Anticoagulation	Citrate
Blood flow rate	250–300 mL/min
Dialysate flow rate	500–800 mL/min
Assessment of the treatment effect	TT, Hemoclot, aPTT

*TT* thrombin clotting time, *aPTT* activated partial thromboplastin time

As a thrombin inhibitor dabigatran affects the final step of the coagulation cascade and thrombin-mediated platelet activation. Interventions that interfere with the coagulation cascade above this level do not appear promising, at least from a theoretical point of view. The application of fresh-frozen plasma or PCC normalized the results of *ex-vivo* coagulation tests or improved the bleeding tendency neither in healthy volunteers given dabigatran nor in dabigatran-treated patients [12, 24]. Three-factor concentrates contain coagulation factors II, IX and X, four-factor concentrates also factor VII.

Compelling data that would suggest the use of activated PCC, which contain activated coagulation factors II and VII are missing. rFVIIa induces the formation of thrombin on the surface of platelets in the absence of tissue factor. To date, there is no evidence that rFVIIa has a significant impact on dabigatran-related coagulation impairment, neither in volunteers nor in bleeding patients.

As a caveat, the possibility of excessive coagulation activation resulting in thrombotic complications including stroke, myocardial infarction or pulmonary embolism must be taken into account when administering coagulation factors, in particularly activated factors.

### Management of acute bleeding (Fig. 1)

**Fig. 1:**
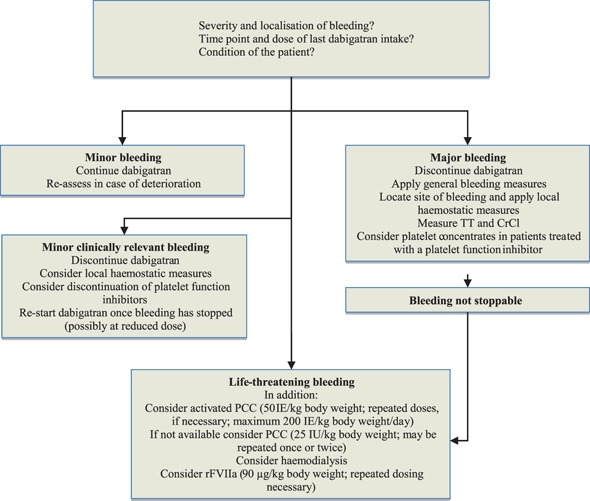
Specific measures for dabigatran-related bleeding

The management of acute bleeding depends upon severity, location and dose and time point of last dabigatran intake.

*Minor bleeding* such as mild nose bleeding, bruises or gum bleeding usually does not necessitate discontinuation of dabigatran treatment. Another medical consultation is required if symptoms deteriorate.

*Minor but clinically relevant bleeding* includes prolonged and/or extensive nose bleeding, large (post-traumatic) haematomas, haematuria or metrorrhagia. In these instances treatment with dabigatran needs to be discontinued and local haemostatic measures should be applied. A medical history to exclude disorders associated with an increased risk of bleeding such as von Willebrand disease, thrombocytopenia or impaired liver and kidney function should be obtained. Drugs affecting platelet function such as aspirin, clopidogrel, non-steroidal anti-inflammatory drugs or selective serotonin and norepinephrine re-uptake inhibitors should be discontinued, unless their use is mandatory. Kidney function should be monitored in patients with renal disease or dehydration. Once bleeding has stopped, treatment with dabigatran can be resumed, possibly at a lower dose.

*Major bleeding* includes gastrointestinal haemorrhage, bleeding requiring transfusion of red blood cells or bleeding associated with a significant decrease in the haemoglobin level, critical organ bleeding (with the exception of the central nervous system; see next paragraph on life-threatening bleeding) or severe posttraumatic haemorrhage. Major bleeding requires discontinuation of dabigatran, rapid location of the bleeding site and local haemostatic measures. Patients receiving a platelet function inhibitor should be given platelet concentrates. The extent of bleeding management is dependent on the severity of bleeding and on the condition of the patient. Dabigatran-sensitive laboratory tests (TT or Hemoclot) and the CrCl allow estimating the duration of the dabigatran effect. If bleeding cannot be stopped by the aforementioned measures, it has to be regarded life-threatening and the following interventions should be applied (see also Fig. 1).

*Life-threatening bleeding* includes bleeding leading to haemodynamic collapse, bleeding after polytrauma, or intracranial haemorrhage. In addition to general intensive-care measures, we recommend administration of an activated PCC (initial dose 50 IE/kg body weight; repeated dosing if necessary; maximal dose 200 IE/kg body weight per day). If an activated PCC is not available, a 4-factor PCC should be given (initial dose 25 IE/kg body weight; may be repeated once or twice). Haemodialysis can be considered taking into account feasibility and risk. Ultimately, rFVIIa (initially 90 µg/kg body weight; repeated dosing) can be given.

### Management of emergency surgery

In case of emergency surgery, dose and time point of last dabigatran intake, extent, type and urgency of surgery and the clinical condition of the patient have to be taken into consideration. Dabigatran treatment has to be discontinued. CrCl has to be measured and a dabigatran-sensitive coagulation assay (TT or Hemoclot) needs to be performed.

If TT is normal, one can assume that there is no effect of dabigatran and dabigatran-specific measures are not useful. A prolonged TT does not correlate with the risk of bleeding. The risk of delaying surgery has to be balanced against the potential risk of dabigatran-associated bleeding. In most cases surgery can be postponed for some hours allowing the dabigatran plasma level to decrease. In rare cases such as in patients with ruptured aortic aneurysm, aortic dissection or polytrauma, results of coagulation assays should not be awaited and the patient should undergo surgery immediately.

In case of intra-operative bleeding, administration of activated PCC can be considered in addition to local haemostatic and general bleeding measures. The possible benefits and the feasibility of haemodialysis should be considered and weighed against its risks. Ultimately, rFVIIa can be given.

### Management of ST-segment elevation myocardial infarction/high-risk non-ST elevation acute coronary syndrome

Dabigatran needs to be discontinued. In patients with ST-segment elevation myocardial infarction or high-risk non-ST elevation acute coronary syndrome, the coronary intervention should be performed according to current guidelines. Specific coagulation tests are not useful, because the urgent need for the intervention precludes waiting for the test results. To minimise the bleeding risk, interventions using a radial approach are preferable.

When an elective percutaneous coronary intervention is performed, dabigatran should be suspended on the day of intervention. Angiography should be performed with or without coronary intervention according to routine protocols.

Patients, who have received a stent and have a persisting indication for oral anticoagulation, should be switched to a vitamin K antagonist. For patients who have received a stent and in whom triple therapy is required, recommendations for dabigatran are lacking. In RE-LY, 38 % of patients received concomitant platelet function inhibitors [25]. In these patients bleeding occurred more frequently both with dabigatran and warfarin as compare with patients given dabigatran or warfarin alone. However, the benefit of dabigatran over warfarin was retained. The combination of dabigatran with a platelet function inhibitor will be recommended in the 2014 ESC guidelines.

### Management of ischaemic stroke

In general, in patients with ischaemic stroke thrombolysis should only be performed in the absence of any dabigatran effect. The use of thrombolytic agents can be considered in patients who have a normal TT (when quantitatively assessed in diluted plasma samples) and/or a normal aPTT.

### Management of overdosing

In the case of dabigatran overdose, close surveillance is recommended along with forced diuresis to decrease the dabigatran levels as quickly as possible. If dabigatran was ingested within the last 2 h (to 4 h), administration of activated charcoal should be considered. Other measures should only be considered in case of bleeding (see chapter on “Acute bleeding”).
